# Spatial and temporal variations and significance identification of ecosystem services in the Sanjiangyuan National Park, China

**DOI:** 10.1038/s41598-020-63137-x

**Published:** 2020-04-09

**Authors:** Wei Cao, Dan Wu, Lin Huang, Lulu Liu

**Affiliations:** 1 Key Laboratory of Land Surface Pattern and Simulation, Institute of Geographic Sciences and Natural Resources Research, Chinese Academy of Sciences, Beijing, 100101 China; 2 Nanjing Institute of Environmental Sciences, Ministry of Ecology and Environment, Nanjing, 210042 China; 3 School of Architecture and Civil Engineering, Chengdu University, Chengdu, 610106 China

**Keywords:** Ecosystem ecology, Ecosystem services

## Abstract

The establishment of the Sanjiangyuan National Park (SNP) favours implementation of strictest ecological protection on the Tibetan Plateau, thus firmly ensuring national ecological security. To understand ecological background in the SNP, spatial and temporal variations of ecosystem, and its services during the period 2000–2015 and significance identification were analysed by using the methods of remote sensing, GIS and model simulation. The results showed that: (1) Area with extremely important ecosystem services accounted for approximately 51.4% of the SNP’s total area, of which extreme importance water regulation, soil conservation and sand fixation regions contributed 15.3%, 13.7% and 22.4%, respectively. (2) The SNP had formed a spatial pattern of ecosystem services with water regulation as core in the eastern part, soil conservation as core in the central part and sand fixation as core in the western part. (3) For the period 2000–2015, water regulation service generally improved in the SNP. Soil conservation service also improved overall; and sand fixation service exhibited a decreasing trend due to reduction in wind speed and vegetation coverage. (4) Climate warming and humidification, combined with the implementation of ecological protection project in the SNP were the primary reasons for ecosystem services improvement. However, grassland degradation had not yet been fundamentally suppressed, and vegetation coverage was still declining in regional areas. For strict protection and sustainable use of the SNP and its natural resources, overall planning and scientific layout should be paid more attention, and classification and subarea protection should be implemented based on natural ecosystem laws.

## Introduction

Ecosystem services are the benefits that human beings obtain from the ecosystem^1^, which are primarily affected by human activity, especially changes in land use and landscape patterns^2,3^. Both the Millennium Ecosystem Assessment (MA) and the Intergovernmental Science-Policy Platform on Biodiversity and Ecosystem Services (IPBES) place great emphasis on the ecosystem services shared by different countries throughout the world^4,5^. Researchers have conducted multiple studies that evaluate ecosystem services as well as their quantification and valuation at different temporal and spatial scales^6–8^. In addition to ecosystem service assessment, ecosystem service flows, supply and demand relationships, trade-offs or synergies among ecosystem services have also been studied^9–11^. The methodology employed in ecosystem services assessment is gradually becoming quantitative and spatialized. At present, strengthening the integration between ecosystem processes and services, and objectively reflecting the formation mechanism of ecosystem services are the new trend in ecosystem service assessment^12^.

National parks are specific terrestrial or marine areas with clear borders that are established by a country and managed to achieve scientific protection and the rational utilization of natural resources. The primary goals of natural parks are to protect large areas of natural ecosystems that are nationally representative and promote harmonious coexistence between humans and nature^13^. Currently, studies on national parks are predominantly conducted from the perspectives of system construction, system innovation, model development, and tourism development, whereas assessments of national parks from an ecological perspective are relatively limited^14–16^. Resource assessment is one of the important methods for the functional division of national parks^17^. Studies on the functional sectorization of national parks began relatively late in China, and evaluations or analyses were mostly limited to a specific year. Due to the influence and changes in the external environment, such as precipitation periodicity, there was some uncertainty whether single-year evaluation results could truly reflect ecological status^18^. Quantitative analysis of spatial and temporal variation in ecosystem patterns and ecosystem services over a certain time period before the establishment of a national park is extremely important for strengthening natural resource management, guiding overall planning and partition control, and exploring ecological compensation mechanisms.

The Sanjiangyuan region is an important part of the ecological security barrier along the Tibetan Plateau in China. Given its fragile ecological environment, the ecosystem has continuously deteriorated over the last several decades under the multiple effects of global climate warming and intensified human activity^19–21^. To highlight the protection of typical representative ecosystems in the Sanjiangyuan source area, strengthen the protection of the “Chinese Water Tower” and emphasize the optimization and reorganization of natural reserves to enhance their connectivity, coordination, and integrity, a pilot national park was proposed for this region in 2015. And the Sanjiangyuan National Park (SNP) was formalized in 2018 and would be officially established in 2020^17^. Scholars have intensively studied ecosystem services of the Sanjiangyuan region, such as tis temporal and spatial distribution of water regulation^22,23^, carbon fixation^24^, biodiversity^25^, grass yield^26^ and vegetation coverage^27^. The research objects are predominantly wetland and grassland ecosystems. Furthermore, studies have been conducted on ecological compensation mechanisms based on ecosystem service value^28^, effects assessment of ecological project^29,30^, changing mechanisms of ecosystem services under the implementation of ecological projects and climate change^31^. However, previous studies have primarily focused on the Sanjiangyuan region or a single basin of the Yangtze River or Huanghe River, which does not present an accurate reflection of the ecological background of the SNP over the past several years.

As China’s first pilot of national park system and the largest national park, how to carry out conservation and management in different zones in the SNP is the biggest problem facing at present. How to develop functional zoning according to the importance of ecosystem structure and services, and then formulate targeted conservation and management measures? The most important thing is to understand the past trend and current situation of ecosystem in the SNP. In this paper, we used geographic information system (GIS), remote sensing (RS), and ecological assessment models to quantitatively analyse the spatial and temporal characteristics of ecosystems and its services in the SNP from 2000 to 2015. We were aiming at figure out the ecological status of the SNP and identify the significance of ecosystem services, which may provide a scientific basis for dividing management zones and performing differentiated protection, as well as developing a top-level design of China’s national parks system.

## Materials and Methods

### Study area

**Figure 1:**
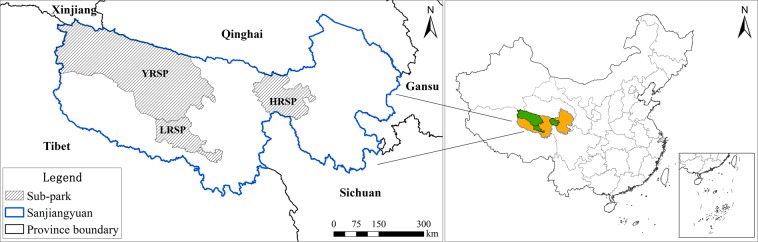
Location of the Sanjiangyuan National Park (SNP). Map created in ArcMap 10.4 (Environmental Systems Resource Institute, ArcMap 10.4 ESRI, Redlands, California, USA).

The SNP consists of the Huanghe River Source Park (HRSP), the Yangtze River Source Park (YRSP), and the Lancang River Source Park (LRSP) (Fig. 1). It covers an area of 12.31 × 10^4^ km^2^, accounting for 31.16% of the total area of the Sanjiangyuan region in North-eastern Tibetan Plateau. Specifically, the HRSP has an area of 1.91 × 10^4^ km^2^ and includes two subzones (the Zaling Lake-Eling Lake, and the Xingxing Hai) of the Sanjiangyuan National Nature Reserve (SNNR). The YRSP has an area of 9.03 × 10^4^ km^2^ and includes the Hoh Xil National Nature Reserve and a subzone (the Suoga-Trama River) of the SNNR. The LRSP has an area of 1.37 × 10^4^ km^2^ and includes two subzones (the Guozongmucha and the Angsai) of the SNNR.

### Data collection and processing

Ecosystem types: Imageries were obtained and interpreted based on multi-source satellite images from Landsat TM/ETM+ , the China-Brazil Earth Resource Satellite (CBERS), and the Beijing-1 environment satellite. Images were pre-processed by radiation calibration, atmospheric correction, and geometric precision correction. The accuracy of the interpretation data reached approximately 95%^32^.

Normalized differential vegetation index (NDVI): The gridded 16-day 250 m NDVI MOD13Q1 products (2000–2015) were obtained from Distributed Active Archive Center, Oak Ridge National Laboratory (ORNL DAAC) (https://modis.ornl.gov/citation.html). The vegetation coverage was derived from the NDVI by dimidiate pixel model^33^ as follow, based on the definition that a pixel of NDVI data could consists of non-vegetation and green vegetation information.

1 $${\rm{V}}{\rm{G}}{\rm{C}}=\frac{NDVI-NDV{I}_{min}}{NDV{I}_{max}-NDV{I}_{min}}$$

In which, NDVI_*max*_ is the value of totally pure vegetation and NDVI_*min*_ is the value of bare land without vegetation.

Meteorological observation data: Meteorological data (precipitation, temperature, wind speed, wind direction, sunshine duration) from 2000 to 2015 were provided by the China Meteorological Data Service Center (CMDSC) (http://data.cma.cn/). The ANUSPLIN interpolation method^34^ was used to interpolate the station observation data into grid data with 1-km spatial resolution.

Terrain data: The DEM data with a resolution of 90 m was derived from Geospatial Data Cloud (http://www.gscloud.cn/).

Soil data: It was collected from the Resource and Environmental Science Data Centre, Chinese Academy of Sciences (http://resdc.cn/). The dataset predominantly included the properties of soil type, soil particle content, and soil organic matter content.

Snow depth data: It was collected from the Environmental and Ecological Science Data Center for West China, National Natural Science Foundation of China (http://westdc.westgis.ac.cn).

### Ecosystem services assessment methods

According to the location of Sanjiangyuan area in China’s ecological barrier, as well as the conservation objectives of the SNP, three important ecosystem services (water regulation, soil conservation and sand fixation) in the SNP are selected for analysis through importance ranking. In addition, as there was a lack of relevant detailed spatial variation data, biodiversity protection was not discussed in this paper.

#### Water regulation

The water regulation capacity (*Q*) of grassland, wetland and forest ecosystems were defined as the difference of water regulation volume between the three ecosystems (*Qe*) and bare land (*Qb*). The water regulation was calculated according to the precipitation storage method^35^ as follows:

2 $${\rm{Q}}={Q}_{e}-{Q}_{b}$$

3 $${Q}_{e}={\rm{A}}\times {\rm{J}}\times {\rm{R}}$$

4 $${\rm{J}}={J}_{0}\times K$$

5 $${\rm{R}}={R}_{0}-{R}_{g}$$

where *Qe* is the water regulation volume of forest, grassland and wetland ecosystems (m^3^); *A* is the ecosystem area (hectare); *J* is the annual output of flow rainfall (mm); *J*_0_ is annual precipitation (mm); *K* is the proportion of annual output of flow rainfall to precipitation amounts; *R* is the profit coefficient of an ecosystem’s decreasing runoff compared with bare land; *R*_0_ is the rainfall runoff rate of bare land under the rainfall-runoff condition; *R*_*g*_ is the rainfall runoff rate of ecosystems under the rainfall-runoff condition. The K value was improved to reflect the spatial difference based on ground surveys, remote sensing data, meteorological data, and several years of river runoff data^36^. The *R*_*g*_ of forest was obtained from relevant literatures, and *R*_*g*_ of grassland was obtained through vegetation coverage^37^.

#### Soil conservation

Soil conservation capacity (*A*_*conservation*_) was defined as the potential capacity of ecosystems to decreasing soil erosion intensity compared to extreme degradation (*A*_*latent*_). Soil erosion intensity was calculated according to the Revised Universal Soil Loss Equation (RUSLE). The formulas are calculated as^38–41^:

6 $${A}_{conservation}={A}_{latent}-{A}_{real}$$

7 $${A}_{real}={\rm{R}}\times {\rm{K}}\times {\rm{L}}\times {\rm{S}}\times {\rm{C}}\times {\rm{P}}$$

8 $${A}_{latent}={\rm{R}}\times {\rm{K}}\times {\rm{L}}\times {\rm{S}}\times {C}_{latent}$$

9 $${\rm{K}}=[2.1\times {10}^{-4}(12-{\rm{OM}}){M}^{1.14}+3.25({\rm{S}}-2)+2.5({\rm{P}}-3)]/100\times 0.1317$$

10 $${\rm{L}}={\left(\frac{\lambda }{22.13}\right)}^{\frac{\beta }{1+\beta }}$$

11 $$\beta =(\frac{sin\theta }{0.0896})/(3.0\cdot sin{\theta }^{0.8}+0.56)$$

12 $$S=\{\begin{array}{c}10.8\,\sin (s)+0.03\,\theta  < 9 \% \\ 16.8\,\sin (s)-0.5\,9\le \,\theta \le 18 \% \\ 21.91\,\sin (s)-0.96\,\theta  > 18 \% \end{array}$$

13 $$C=\{\begin{array}{c}1\,VGC=0\\ 0.6508-0.3436lgVGC\,0 < VGC\le 78.3 \% \\ 0\,VGC > 78.3 \% \end{array}$$

where *A* is the soil erosion module (ton/hectare·yr). *R* is the rainfall erodibility factor (MJ·mm/hectare·hour·yr), which was calculated by a rainfall erosivity model using daily rainfall amounts. *K* is the soil erodibility factor (ton·hour/(MJ·mm)), which was estimated by the Nomo model^39^ based on the 1:1 million soil data. *L*(m) is the slope length factor and *S*(%) is the slope factor, which are calculated by the core algorithm^40^ improved by considering runoff barriers of land surface parameters. *C* is the vegetation cover factor that was calculated based on VGC, and time intervals for both rainfall erodibility and fractional coverage factors determined for 16 days to avoid time asynchronism and to reduce errors^41^. *P* is the erosion control practice factor with a value range of 0–1, which was mainly determined from relevant literature and expert consultations, with *P* values of grassland, forest and bare land set at 1, paddy fields at 0.15, dry land at 0.352, settlement at 0.01, and wetland/water bodies at 0.

#### Sand fixation

Sand fixation capacity (*SL*) was defined as the potential capacity of ecosystems (*SLe*) to decreasing wind erosion intensity compared to bare soil conditions (*SLb*). Wind erosion intensity was calculated using the Revised Wind Erosion Equation (RWEQ) as follows^42^:

14 $${\rm{SL}}=S{L}_{e}-S{L}_{b}$$

15 $${\rm{SL}}={Q}_{x}/x$$

16 $${Q}_{x}={Q}_{max}\left[1-{e}^{{\left(\frac{x}{s}\right)}^{2}}\right]$$

17 $${Q}_{max}=109.8(WF\cdot EF\cdot SCF\cdot K{\prime} \cdot COG)$$

18 $$s=150.71{(WF\cdot EF\cdot SCF\cdot K{\prime} \cdot COG)}^{-0.3711}$$

19 $$WF=\frac{{\sum }_{i=1}^{N}W{S}_{2}{(W{S}_{2}-W{S}_{t})}^{2}\times {N}_{d}\rho }{N\times g}\times SW\times SD$$

20 $$EF=\frac{29.09+0.31{S}_{a}+0.17{S}_{i}+0.33\frac{{S}_{a}}{{C}_{l}}-2.59OM-0.95CaC{O}_{3}}{100}$$

21 $$SCF=\frac{1}{1+0.0066{({C}_{l})}^{2}+0.021{(OM)}^{2}}$$

where *SLe* is the annual wind erosion module (ton/hectare/yr); *x* is the field length (m); *Q*_*x*_ is the sand flux at length *x* of the field (kg/m); *Q*_*max*_ is the maximum transport capacity (kg/m); *s* is the critical field length (m); *WF* is the climate factor (kg/m), which reflects the combined effect of the wind factor, the air density, acceleration due to gravity, the soil wetness factor, the snow cover factor on the wind erosion. The wind factor and the soil wetness factor used the station’s daily data obtained from the CMDSC. The snow cover factor was calculated using the long-term snow depth dataset. *EF* is the soil erodibility factor, and *SCF* is the soil crust factor. *K*′ is the soil roughness factor, which was measured by using a roller chain method and measured the sand and grassland roughness factors as having values of 0.96 and 0.69 respectively. *COG* is the vegetation cover factor that includes three factors of flat residues, standing residues, and growing vegetation, and is calculated by the fractional vegetation cover depending on the effect of either withered or growing vegetation on wind erosion by field observation combining visual estimation and traditional photographic recording.

### Analysis methods

#### Variation trend analysis

The annual variation trends for water regulation, soil conservation, sand fixation, vegetation coverage, and meteorological factors (annual precipitation, temperature and wind speed) were calculated according to the least square method^43^. The formula is expressed as:

22 $${\rm{S}}=\frac{{\rm{n}}\times {\sum }_{{\rm{i}}=1}^{{\rm{n}}}{\rm{i}}\times {{\rm{X}}}_{{\rm{i}}}-{\sum }_{{\rm{i}}=1}^{{\rm{n}}}{\rm{i}}{\sum }_{{\rm{i}}=1}^{{\rm{n}}}{{\rm{X}}}_{{\rm{i}}}}{{\rm{n}}\times {\sum }_{{\rm{i}}=1}^{{\rm{n}}}{{\rm{i}}}^{2}-{({\sum }_{{\rm{i}}=1}^{{\rm{n}}}{\rm{i}})}^{2}}$$

where *S* is the variation trend; *X*_*i*_ is the value of the variables for each year i; n is the total number of years.

#### Significance identification of ecosystem services

In light of the significance of ecosystem services is likely to play in linking conservation activities and human benefits, identification of the significance of ecosystem services were processed by measuring, modelling and mapping single ecosystem services, and then systematically integrated to providing scientifically baselines. Significance identification of single ecosystem service was used according to the Guidelines for ecological conservation redline delineation^44^, which provided the classification standards for ecosystem service. Taking soil conservation as an example, capacity values of ecosystem soil conservation were sorted and accumulated from the high to the bottom. The values that the accumulated amount accounted for 50% and 80% of the total amount were considered to be demarcation points for significance identification. According to the two demarcation points, significance of soil conservation was reclassified into the following three levels in ArcGIS: extremely important region, important region and generally important region.

## Results

### Variation patterns of ecosystem types in the SNP

**Figure 2:**
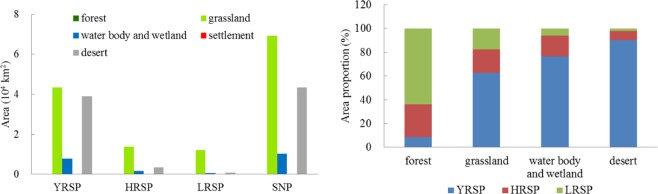
Area (left) and proportion (right) of different ecosystem types in various sub-parks of the SNP.

In 2015, the SNP was dominated by grassland, desert, water body and wetland ecosystems with areas of 6.90 × 10^4^ km^2^, 4.34 × 10^4^ km^2^, and 1.04 × 10^4^ km^2^, respectively, which accounted for 56.2%, 35.2%, and 8.4% of the national park’s total area (Fig. 2).

The YRSP was dominated by grassland and desert ecosystems, accounting for 48.0% and 43.2% of its area. The grassland ecosystem was predominantly distributed in the southeast, whereas the desert ecosystem was predominantly distributed in the northwest. There were also numerous lakes and wetlands, which accounted for approximately 8.8% of its area, and 76.7% of the water body and wetland ecosystems in the SNP (Fig. 2).

The HRSP was dominated by grassland ecosystem, which accounted for approximately 72.5% of its area, followed by desert ecosystem, constituting approximately 17.8%. There were also plateau lakes (represented by Zhaling Lake and Eling Lake) and wetlands, which accounted for approximately 17.3% of the water body and wetland ecosystems in the SNP.

The grassland ecosystem in the LRSP comprised 88.1% of the park’s area. The desert, water body and wetland ecosystems constituted less than 10%. In addition, a small number of forest ecosystem distributed in the southern part of the LRSP, accounted for approximately 63.8% of the forest in the SNP.

**Figure 3:**
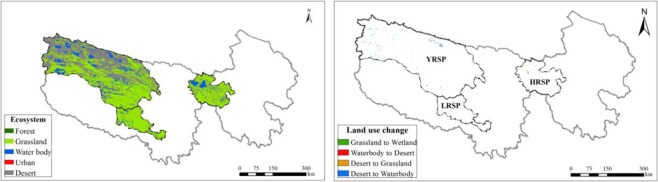
Spatial distribution of ecosystem types in 2015 of the SNP (left) and ecosystem changes from 2000 to 2015 (right). Map created in ArcMap 10.4 (Environmental Systems Resource Institute, ArcMap 10.4 ESRI, Redlands, California, USA).

Spatial distribution of ecosystem types in 2015 and ecosystem changes of the SNP from 2000 to 2015 were shown in Fig. 3. In general, ecosystem patterns changed not obvious during this period, which was mainly occurred in desert, water body and wetland ecosystems. Area of water body and wetland ecosystem increased by 416.68 km^2^, while desert area decreased by 356.75 km^2^. In spatial, ecosystem types changed mainly occurred in the YRSP and HRSP (Fig. 3).

### Spatial and temporal variations of ecosystem services in the SNP

#### Water regulation

**Figure 4:**
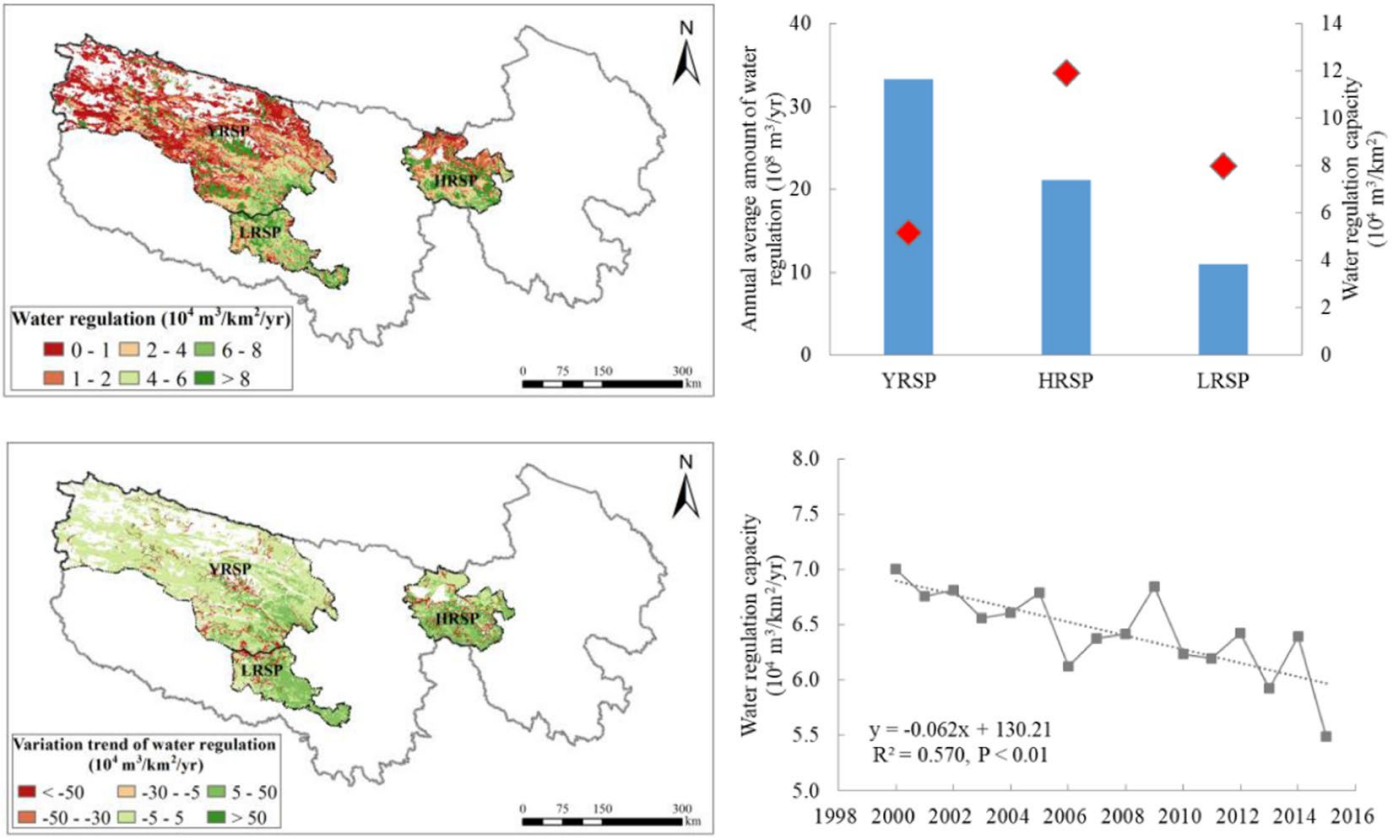
Spatial distribution of water regulation service in the SNP (left upper) and its changes from 2000 to 2015 (left bottom), annual average amount of water regulation and its capacity in the different sub-parks (the bars means water regulation amount and the diamonds means water regulation capacity) (right upper) and interannual variation of water regulation capacity (right bottom). Map created in ArcMap 10.4 (Environmental Systems Resource Institute, ArcMap 10.4 ESRI, Redlands, California, USA).

The annual average amount of water regulation in the SNP was approximately 6.54 billion m^3^/yr during the period of 2000 to 2015. Water regulation capacity was approximately 0.068 million m^3^/km^2^ during this period. It was higher in the southeast and lower in the northwest (Fig. 4). The annual average amount of water regulation in the YRSP was the highest (3.33 billion m^3^/yr), followed by the HRSP (2.12 billion m^3^/yr), and the lowest in the LRSP (1.09 billion m^3^/yr). Water regulation capacity of the HRSP was the highest (0.12 million m^3^/km^2^), followed by the LRSP (0.08 million m^3^/yr), and the lowest in the YRSP (0.05 million m^3^/km^2^) (Fig. 4).

The annual amount of water regulation in the SNP exhibited a decreasing trend of −0.056 billion m^3^/yr, featuring fluctuations during the period of 2000 to 2015. The changing trend of water regulation capacity was approximately −617 m^3^/km^2^ during this period (Fig. 4). In particular, variation trend for water regulation amount increased approximately 1.33 million m^3^/yr in the HRSP. However, it decreased by −40.74 million m^3^/yr and −16.09 million m^3^/yr in the YRSP and the LRSP, respectively. Approximately 84.5% of the SNP exhibited an increasing trend of annual water regulation amount, and only 14.6% of the region exhibited a decrease. For the three different sub-parks, the regions with increased water regulation amount all exceeded 80% in each sub-park’s area. However, due to the small increased magnitude, annual amount of water regulation in the entire region still exhibited a decreasing trend.

#### Soil conservation

**Figure 5:**
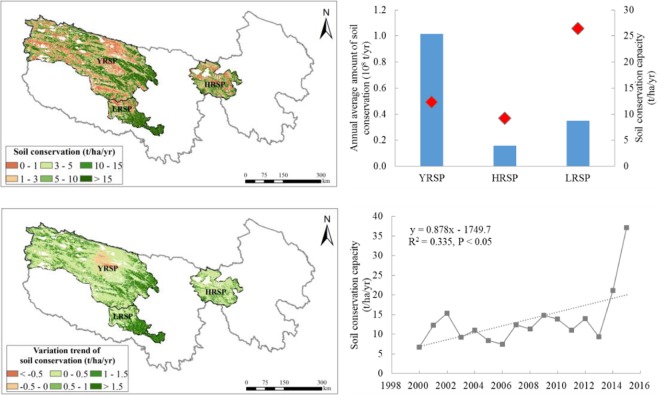
Spatial distribution of soil conservation service in the SNP (left upper) and its changes from 2000 to 2015 (left bottom), annual average amount of soil conservation and its capacity in the different sub-parks (the bars means soil conservation amount and the diamonds means soil conservation capacity) (right upper) and interannual variation of soil conservation capacity (right bottom). Map created in ArcMap 10.4 (Environmental Systems Resource Institute, ArcMap 10.4 ESRI, Redlands, California, USA).

The annual average amount of soil conservation in the SNP was 0.15 billion t/yr, and 13.50 t/hectare/yr per unit area from 2000 to 2015. The soil conservation capacity was higher in the central part and low in the northwest. The annual average amount of soil conservation in the YRSP was the highest (0.10 billion t/yr), followed by the LRSP (0.03 billion t/yr), and the lowest in the HRSP (0.02 billion t/yr). Soil conservation capacity of the LRSP was the highest (26.4 t/hectare/yr), followed by the YRSP (12.3 t/hectare/yr), and the lowest in the HRSP (9.2 t/hectare/yr) (Fig. 5).

The annual amount of soil conservation in the SNP exhibited an increasing trend of 9.87 million t/yr, featuring fluctuations during the period of 2000 to 2015. The trend of soil conservation per unit area was approximately 0.88 t/hectare/yr (Fig. 5). In particular, it increased in the three sub-parks. The increasing was greatest in the YRSP (approximately 5.70 million t/yr), followed by the LRSP (3.31 million t/yr), and smallest in the HRSP (0.86 million t/yr). For the soil conservation capacity, it was increasing largest in the LRSP by approximately 2.52 t/hectare/yr. The trends in the YRSP and the HRSP were comparable by 0.69 t/hectare/yr and 0.50 t/hectare/yr, respectively. The amount of soil conservation exhibited a rising trend in more than 95% of the entire region. Specifically, it exhibited a rising trend in 94.4% of the YRSP, as well as 98.6% and 99.5%, respectively, in the HRSP and the LRSP.

#### Sand fixation

**Figure 6:**
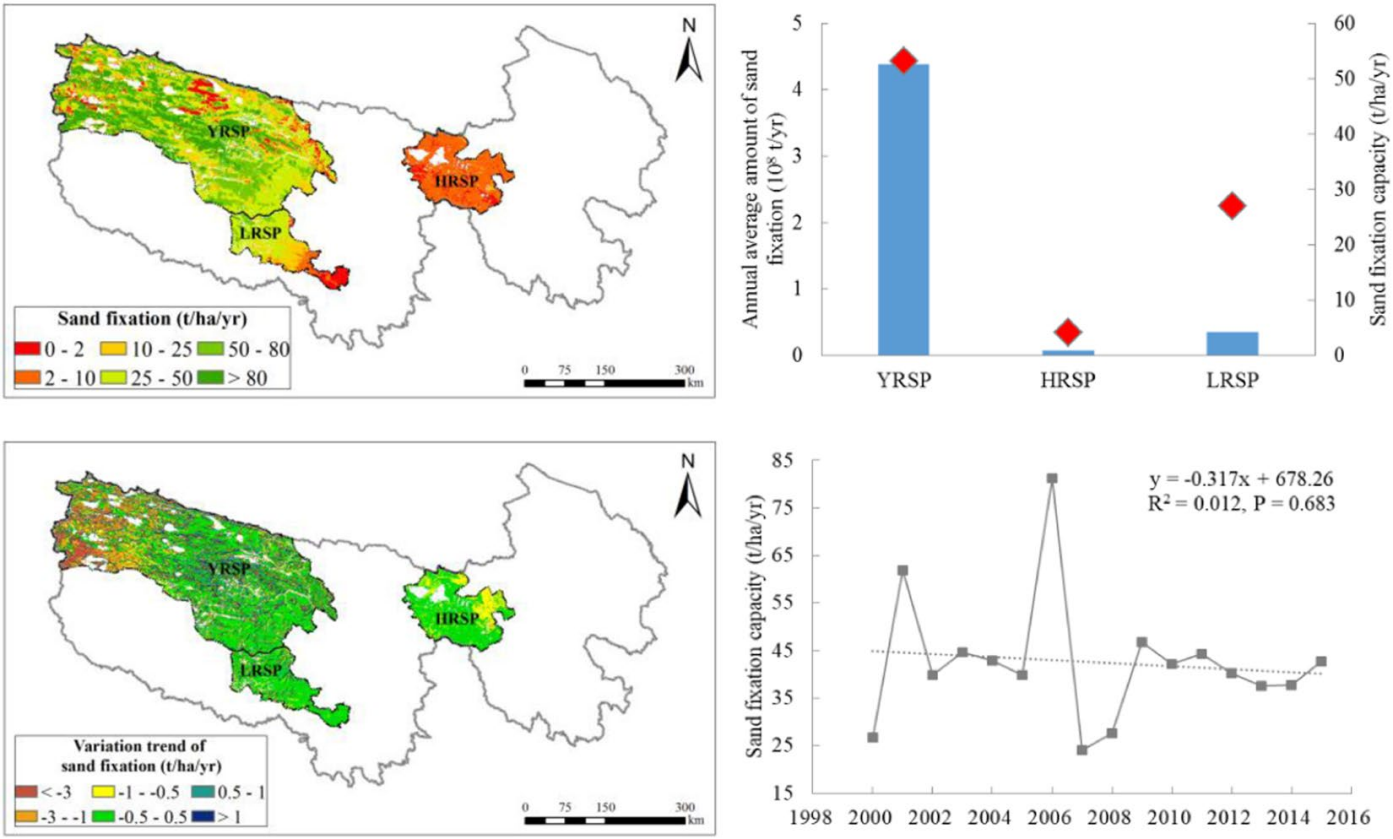
Spatial distribution of sand fixation service in the SNP (left upper) and its changes from 2000 to 2015 (left bottom), annual average amount of sand fixation and its capacity in the different sub-parks (the bars means sand fixation amount and the diamonds means sand fixation capacity) (right upper) and interannual variation of sand fixation capacity (right bottom). Map created in ArcMap 10.4 (Environmental Systems Resource Institute, ArcMap 10.4 ESRI, Redlands, California, USA).

The annual average amount of sand fixation in the SNP was 0.48 billion t/yr, and 42.6 t/hectare/yr per unit area from 2000 to 2015. The sand fixation capacity was higher in the west and lower in the east. The annual average amount of sand fixation in the YRSP was the highest (0.43 billion t/yr), followed by the LRSP (0.04 billion t/yr), and the lowest in the HRSP (0.01 billion t/yr). Sand fixation capacity of the YRSP was the highest (53.1 t/hectare/yr), followed by the LRSP (27.0 t/hectare/yr), and the lowest in the HRSP (4.1 t/hectare/yr) (Fig. 6).

During the period 2000–2015, amount of sand fixation in the SNP exhibited a decreasing trend with fluctuations, and the annual variation trend was approximately −3.56 million t/yr. The variation trend of sand fixation per unit area was approximately −0.32 t/hectare/yr (Fig. 6). Sand fixation capacity in both the YRSP and the HRSP decreased by −0.36 t/hectare/yr. There was a relatively small change in the LRSP. The regions with relatively large-amplitude declines were primarily concentrated in the western part of the YRSP and east of the HRSP. Approximately 39.3% of the SNP exhibited an increasing trend of annual sand fixation amount, and approximately 56.0% of the region exhibited a decrease.

### Comprehensive evaluation of ecosystem services

**Figure 7:**
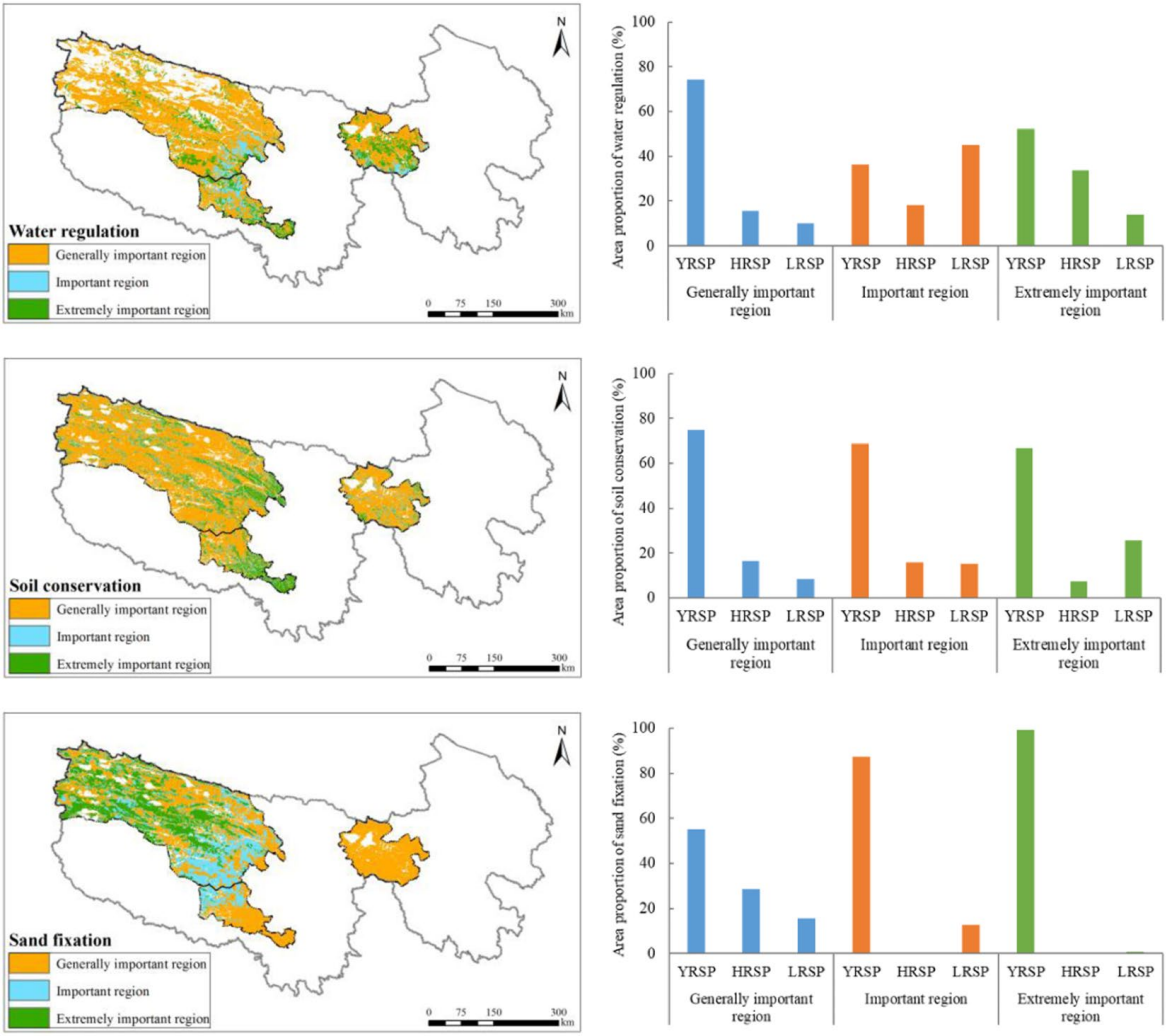
Significance classification of ecosystem water regulation, soil conservation, sand fixation services in the SNP (left) and area proportion of the different sub-parks (right). Map created in ArcMap 10.4 (Environmental Systems Resource Institute, ArcMap 10.4 ESRI, Redlands, California, USA).

Area proportion of the generally important region for water regulation service comprised 74.6% of the SNP, predominantly distributed in the north-western part of the park. In particular, it was the highest in the YRSP, constituting 74% of its area, 16% in the HRSP, and 10% in the LRSP. The important region for water regulation service comprised 10.1% of the SNP, predominantly distributed in the central part of this park. In particular, it was the largest (45%) in the LRSP, followed by 36% in the YRSP, and18% in the HRSP. The extremely important region for water regulation service comprised 15.3%, predominantly distributed in the central and southern parts of the YRSP, central part of the HRSP, and northern and southern parts of the LRSP. In particular, it was the largest (52%) in the YRSP, followed by the HRSP (34%), and 14% in the LRSP (Fig. 7). From 2000 to 2015, the annual amount of water regulation in the extremely important regions declined considerably by approximately −69.69 million m/yr, whereas there were increasing trends in both the generally important and important regions.

The generally important region for soil conservation service comprised 73.5% of the SNP, predominantly distributed in the northwest part of the park. In particular, it was the highest in the YRSP, constituting 75% of its area, 17% in the HRSP, and it was the lowest in the LRSP by 8%. The important region for soil conservation service comprised 12.8% of the SNP, predominantly distributed in the central part of the region. In particular, it was the largest in the YRSP, constituting 69% of its area, 16% in the HRSP, and 15% in the LRSP. The extremely important region for soil conservation service comprised 13.7%, predominantly distributed in the middle eastern part of the YRSP and southern part of the LRSP. In particular, it was the largest (67%) in the YRSP, followed by the LRSP (26%), and 8% in the HRSP (Fig. 7). The soil conservation amount in the extremely important regions demonstrably increased by approximately 5.67 million t/yr. It was also increased in both the generally important and important regions.

The generally important region for sand fixation service comprised 53.0% of the SNP, predominantly distributed in the HRSP, southern part of the LRSP and north central part of the YRSP. In particular, it was the highest in the YRSP, constituting 55% of its area, 29% in the HRSP, and 16% in the LRSP. The important region for sand fixation service comprised 24.6% of the SNP, predominantly distributed in southern part of the YRSP and northern part of the LRSP. In particular, it was the largest in the YRSP, constituting 87% of its area, and 13% in the LRSP. The extremely important region for sand fixation service comprised 22.4%, predominantly distributed in central and western regions of the YRSP. In particular, it was the largest (99%) in the YRSP, followed by the LRSP (1%) (Fig. 7). The average variation of sand fixation amount in the extremely important region exhibited a declining of −3.55 million t/yr. The trend declined slightly in the generally important region and increased in the important region.

**Figure 8:**
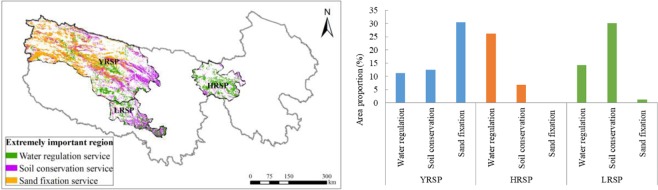
Spatial distribution of the extremely important region for ecosystem services in the SNP (left) and area proportion of the different sub-parks (right). Map created in ArcMap 10.4 (Environmental Systems Resource Institute, ArcMap 10.4 ESRI, Redlands, California, USA).

The extremely important region for ecosystem services comprised 51.4% of the SNP. In which, it were 15.3%, 13.7% and 22.4% for the YRSP, the HRSP and the LRSP, respectively. Among the extremely important regions, the highest water regulation capacity was in the HRSP (0.36 million m^3^/km^2^), the highest soil conservation capacity was in the LRSP (68.4 t/hectare/yr), the highest sand fixation capacity was in the YRSP (95.1 t/hectare/yr) (Fig. 8).

From the sub-park perspective, the extremely important regions accounted for 32.9% of the HRSP, of which extremely important regions of water regulation service was the highest (26.0%). It is evident that the core ecosystem service of the HRSP during the study period was water regulation, which was primarily provided by wetland and grassland ecosystems. The extremely important region accounted for 45.6% of the LRSP, of which soil conservation service was the highest (29.9%). The core ecosystem service of the LRSP was soil conservation, which was primarily provided by forest and grassland ecosystems. The extremely important regions accounted for 54.1% of the YRSP, of which sand fixation service was the highest (30.5%). Finally, the core ecosystem service of the YRSP was sand fixation, which was primarily provided by desert and grassland ecosystems. Therefore, the SNP formed a spatial pattern of ecosystem services with water regulation as core in the eastern part, soil conservation as core in the central part and sand fixation as core in the western part (Fig. 8).

## Discussion

### Factors influencing the fluctuations of ecosystem services

**Figure 9:**
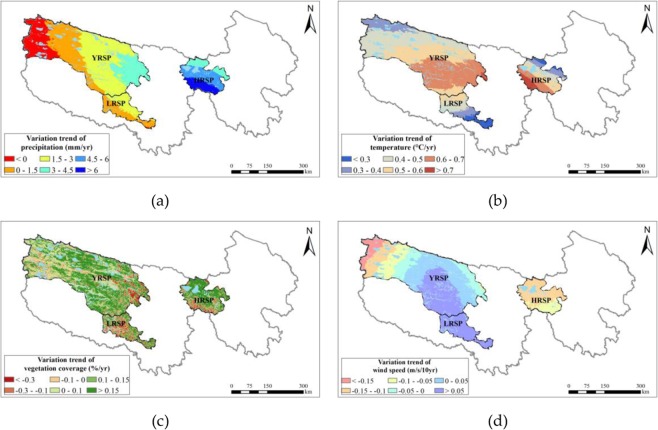
Spatial distribution of variation trends for annual precipitation (**a**), temperature (**b**), vegetation coverage (**c**), and wind speed (**d**) in the SNP during the period 2000 to 2015. Map created in ArcMap 10.4 (Environmental Systems Resource Institute, ArcMap 10.4 ESRI, Redlands, California, USA).

The warming and humidification facilitated by regional climate change combined with the implementation of ecological protection project in the SNP were the primary reasons for the water regulation and soil conservation improvement. From 2000–2015, the overall annual mean temperature and precipitation in the SNP increased by 0.05 °C/yr and 2.10 mm/yr, respectively. In particular, the increasing was highest in the HRSP by 0.053 °C/yr and 5.26 mm/yr, and 0.053 °C/yr and 1.56 mm/yr in the YRSP, respectively. It was not significant in the LRSP by 0.039 °C/yr and 1.40 mm/yr, respectively (Fig. 9a,b). Climate warming and humidification facilitated a return of vegetation to its green stage and prolonged the growing period, further increasing the vegetation coverage^45^.

In addition, a series of ecological conservation and restoration projects were implemented in the Sanjiangyuan region. From 2004 to 2012, the cumulative amount of grazing land returned to grassland was 6.3122 million hectare and 0.4234 million hectare of mountain land was closed for vegetation recover. Furthermore, 0.1846 million hectare of black beach soil was restored, 0.0441 million hectare of land was managed to prevent desertification, and 7.8541 million hectare of grassland was protected from rodents^18^. Under the combined effects of climate change and ecological engineering, grassland degradation in the SNP was contained. Vegetation coverage in the SNP exhibited an increasing trend with fluctuations of 0.005%/10 yr in the past 15 years. In particular, an increasing rate of 0.023%/10 yr was most evident in the HRSP, followed by the YRSP (0.016%/10 yr), and 0.005%/10 yr in the LRSP (Fig. 9c). The ecosystem status of this region improved remarkably.

Water regulation was mainly common affected by precipitation and vegetation coverage in the SNP. Although they both showed increasing trends as a whole from 2000 to 2010, vegetation coverage was still decreased in local areas, which resulted in the decrease of water regulation amount.

The decline in sand fixation was primarily caused by a decrease in wind speed and local reductions in vegetation coverage. The wind speed in the SNP declined at a rate of −0.01 m/s/10 yr from 2000 to 2015 (Fig. 9d), which had large fluctuations between 2005 and 2008. Decreased wind speed reduced the potential wind erosion and caused a reduction in sand fixation. Moreover, by coupling vegetation coverage data and spatial variability data of sand fixation, sand fixation reduction was found to have predominantly occurred in the regions with reduced vegetation coverage, which was most evident in the northwest region of the YRSP.

### Partition management of the SNP

Ecosystem services in the SNP exhibited an overall improvement from 2000 to 2015. However, sand fixation and water regulation in the extremely important region exhibited obvious decreasing trends. Grassland degradation had not yet been completely restored and required further conservation and restoration. Vegetation coverage was still decreased in local areas. Restoration of vegetative root layers was extremely slow, especially in the western region with dry climate conditions. The warming trend was evidently higher than that of wetting, Liu *et al*. predicted that potential evapotranspiration would increase^27^, thus weakening the warming and humidification trend in the Sanjiangyuan region, which would promote a warming and drying climate and further inhibit vegetation growth^46^. In addition, although the increasing vegetation coverage was evident in the HRSP, the improvement was only exhibited in terms of growth but not community structure. Furthermore, warming would accelerate the melting of glaciers, permanent snow and frozen soil. The ecological security is severely threatened when regional ecosystem’s equilibrium is broken. Thus, to achieving overall planning conservation goals for the SNP, a longterm process remains need.

According to the general plan for the establishment of the national park system in China, and the guidance on the establishment of the natural reserve system with the National Park, in the process of the planning and construction of the SNP, different management strategies must be applied according to the primary ecosystem types, core ecosystem services, and its significance, and the local climate conditions. In the HRSP, the focus should be on the protection of grassland and wetland ecosystems, and strict protection mode for the extremely important region. For the grasslands and wetlands of the important and generally important regions, ecological measures such as restricting grazing, returning pasture to grasslands, balancing grass production and livestock, and artificially seeding grass could be implemented, and degraded grasslands should be restored. Furthermore, subsidy and incentive policies should be implemented to promote ecological protection. Additionally, ecological organic animal husbandry could be moderately developed. Thus, vegetated areas and coverage would be increased, which could enhance the capacity of water regulation and soil conservation.

For the YRSP, besides grassland restoration, the protection of snowy mountains, glaciers and deserts should be emphasized. In the extremely important region, the function of natural ecosystems must be maintained, by strictly restricting human activities around glaciers and snowy mountains to prevent the exposure of soil and rock. According to the natural conditions of desert areas, grassland production and the biological control of sand, appropriate construction of a crosscutting protective zone close to the sand belt could be adopted to further improve sand fixation capacity in this region. In the important and generally important regions, ecological organic animal husbandry and sand industry could be moderately developed by strictly implementing some balance between grassland and livestock, and desert land management.

For the LRSP, beside grasslands, we should focus on the protection of forest ecosystems. In the extremely important region, the comprehensive protection of public welfare forest compensation, closed forests, forest fire prevention and pest control should be the key measures. In the important and generally important regions, afforestation and the cultivation of middle-aged and young forests could be moderately conducted to enlarge forest coverage, improve stand structure and enhance forest quality and ecological benefit, which could further improve soil conservation and water regulation services.

## Conclusions

This paper analysed ecosystem types and the spatial differentiation of ecosystem services in the SNP, and revealed the temporal and spatial variation trends of ecosystem services during the period 2000 to 2015, and classified the significance of each ecosystem service. The study helps to establish the ecological background of the SNP and scientifically delineates management zones. The primary conclusions were as follows:

1. Ecosystem types of the SNP were primarily grassland, desert, water body and wetland ecosystems. In particular, the YRSP was dominated by grassland and desert ecosystems; the HRSP was dominated by grassland ecosystem; and the LRSP was dominated by grassland ecosystem. A small amount of forest ecosystem was distributed in the south part of the region.
2. Water regulation capacity was higher in the southeast and lower in the northwest. It was the highest in the HRSP, followed by the LRSP. Soil conservation capacity was high in the central part and low in the northwest. It was the highest in the LRSP, followed by the YRSP. Sand fixation capacity was higher in the west and lower in the east. It was the highest in the LRSP, followed by the HRSP.
3. The SNP had formed a spatial pattern of ecosystem services with water regulation as core in the east, soil conservation as core in the central and sand fixation as core in the west.
4. In the period 2000–2015, water regulation service in the SNP improved overall. Proportion of increased areas was relatively large but the magnitude was low. Soil conservation service was also improved overall; and sand fixation service exhibited a decreasing trend.

Further research would focus on the improvement of ecosystem services and the coordinated utilization of water and soil resources in the alpine region. Through the organic combination of ecosystem pattern-process-service, anthropogenic and naturally driven mechanisms of ecosystem service maintenance could be more intensively studied. Research findings could provide a scientific basis for the permanent protection of authenticity and integrity of ecosystems among mountains, water bodies, forests, farmlands, lakes and grasslands in the SNP.

## Data Availability

Data are accessible through Distributed Active Archive Center, Oak Ridge National Laboratory (https://modis.ornl.gov/citation.html), China Meteorological Data Service Center (http://data.cma.cn/), Geospatial Data Cloud (http://www.gscloud.cn/) Resource and Environmental Science Data Centre, Chinese Academy of Sciences (http://resdc.cn/) and Environmental and Ecological Science Data Center for West China, National Natural Science Foundation of China (http://westdc.westgis.ac.cn).
